# Effect of Endovascular Treatment on Systemic Vascular Resistance in Patients with Lower-Limb Peripheral Artery Disease

**DOI:** 10.3400/avd.oa.20-00086

**Published:** 2020-12-25

**Authors:** Hidetsugu Nomoto, Toshihiro Nozato, Shu Yamashita, Masahito Suzuki, Tomoyo Sugiyama, Tetsuo Oumi, Masakazu Ohno, Shigeo Shimizu, Takashi Ashikaga, Yasuhiro Satoh

**Affiliations:** 1 Department of Cardiovascular Medicine, Ome Municipal General Hospital, Ome, Tokyo, Japan; 2 Department of Cardiovascular Medicine, Japanese Red Cross Musashino Hospital, Musashino, Tokyo, Japan; 3 Department of Cardiovascular Medicine, Tokyo Medical and Dental University, Tokyo, Japan; 4 Department of Cardiovascular Medicine, Kameda General Hospital, Kamogawa, Chiba, Japan; 5 Department of Cardiovascular Medicine, National Hospital Organization, Disaster Medical Center, Tachikawa, Tokyo, Japan; 6 Department of Cardiovascular Medicine, Tsuchiura Kyodo General Hospital, Tsuchiura, Ibaraki, Japan; 7 Department of Cardiovascular Medicine, Hiratsuka Kyosai Hospital, Hiratsuka, Kanagawa, Japan

**Keywords:** peripheral artery disease, hypertension, endovascular treatment, noninvasive continuous cardiac output monitoring, antihypertensive

## Abstract

**Objective:** Endovascular treatment (EVT) for lower-limb peripheral artery disease patients reduces blood pressure (BP) and improves prognosis. This study retrospectively examined hemodynamics during EVT to clarify the mechanism.

**Materials and Methods:** Systemic vascular resistance (SVR) was measured using a noninvasive continuous cardiac output monitoring system during EVT. Furthermore, ankle brachial index was measured before and after EVT.

**Results:** The study included 88 lesions of 56 patients (hypertension in 98%). SVR significantly decreased from 2409.1±746.8 dynes·s·cm^−5^ to 2033.7±635.0 dynes·s·cm^−5^ (p<0.0001). The difference in SVR before and after EVT was significantly greater in the Fontaine IV group than in the Fontaine IIa group (554.7±406.6 dynes·s·cm^−5^ vs. 312.9±245.7 dynes·s·cm^−5^, p=0.0151). The change in SVR was correlated with a change in mean BP in the upper limb (p=0.0026). When the change in pressure gradient between the upper limb and the diseased lower limb was large, mean BP of the upper limb significantly decreased (p=0.0022).

**Conclusion:** EVT can reduce SVR and BP by canceling the pressure gradient between central BP and diseased lower-limb BP.

## Introduction

Peripheral artery disease (PAD) is a worldwide problem, and the number of affected patients is currently increasing.^1)^ Cigarette smoking, diabetes mellitus, hypertension, and dyslipidemia are well-known risk factors of PAD. High blood pressure (BP) affects the formation of lower limb and systemic atherosclerosis and, in turn, atherosclerotic arteries can exacerbate hypertension. Patients with PAD frequently report cardiovascular complications and poor prognosis.^2,3)^ Significant stenosis due to advanced arteriosclerosis produces a pressure gradient (PG) between the central and peripheral sides of the lower limbs, resulting in high BP at the central side (i.e., aorta and its main branches, upper limb, brain), pressure overload to the heart and other organs, and hypoperfusion of the diseased lower limbs. Successful endovascular treatment (EVT) improves the quality of life and hemodynamic status of patients with lower-limb PAD. We have recently shown that systemic BP improvements after EVT were related to patient prognosis.^4)^ However, the mechanism of BP reduction by EVT is unknown. Therefore, we examined hemodynamic status, change in BP, and systemic vascular resistance (SVR) using a noninvasive continuous cardiac output monitoring system during EVT for patients with PAD.

## Materials and Methods

### Study design and patient population

This retrospective study included patients with consecutive lower-limb PAD, who underwent EVT, and measured hemodynamic status using an AESCULON mini® noninvasive continuous cardiac output monitoring system (OSYPKA MEDICAL and Heiwa Bussan, Tokyo, Japan) between June 2014 and February 2017 at the National Hospital Organization, Disaster Medical Center, Tokyo, Japan. Patients with only a below-knee lesion were excluded from this study. All data were retrospectively collected. Lower-limb PAD severity was determined according to Fontaine classification. Study participants were surveyed for lesion area, position, length, Trans-Atlantic Inter-Society Consensus (TASC) II classification, hypertension, diabetes mellitus, dyslipidemia, chronic kidney disease, hemodialysis, coronary artery disease, cerebral vascular disease, and smoking habits. Patients were deemed to have hypertension if they had a history or were being treated with antihypertensive drugs. Diabetes mellitus was defined based on the World Health Organization criteria or treatment for the condition. Dyslipidemia was defined as a serum total cholesterol level >220 mg/dL or being treated for the condition. Chronic kidney disease was defined as an estimated glomerular filtration rate (eGFR) <60 mL/min/1.73 m^2^ or the presence of a kidney abnormality such as proteinuria. Hemoglobin, platelet, albumin, blood urea nitrogen, serum creatinine, uric acid, total cholesterol, low-density lipoprotein cholesterol, high-density lipoprotein cholesterol, triglyceride, hemoglobin A1c (National Glycohemoglobin Standardization Program, %), brain natriuretic peptide, and left ventricular ejection fraction using echocardiography were measured before EVT. The eGFR was calculated using the Modification of Diet in Renal Disease study equation coefficients modified for Japanese patients.^5)^ The ankle brachial index (ABI) was measured on the day before and within 1 week after EVT in all patients. Cardiac output, stroke volume, and SVR were measured during EVT using the AESCULON mini® noninvasive continuous cardiac output monitoring system. This device can measure stroke volume using electrical velocimetry, a technique of measuring stroke volume that captures a change in conductivity of the orientation change of erythrocytes flowing through the aorta. It requires the attachment of four electrocardiographic electrodes to the body and has been proven comparable to the thermodilution method.^6)^ SVR and cardiac output were calculated by stroke volume, non-invasive BP measured in the upper limb, right atrial pressure (uniformly 5 mmHg), and heart rate. Mean BP was calculated using the equation: (systolic BP−diastolic BP)/3+diastolic BP.

### Ethics

This study complied with the Declaration of Helsinki, and the ethical review board of the National Hospital Organization, Disaster Medical Center approved the study protocol (reference number: 2015-10). Informed consent was obtained from all patients or their family members before enrollment.

### Procedural details

The interventional strategy and device used were chosen by the operator. In principle, the stent was implanted for aortoiliac lesions. For femoral stenotic lesions, balloon expansion was first used. If major dissection occurred, optional stenting was performed. In cases with femoral chronic total occlusion, primary stenting was used. All patients started dual antiplatelet therapy 1 month in advance and were continuously treated after EVT. In all cases, no additional vasodilator was administered on the day of EVT.

### Statistical analysis

All data are represented as mean±standard deviation, mean±standard error, or median (range or 25%–75% interquartile range). The paired t-test and Wilcoxon signed-rank test were used to examine changes from baseline in hemodynamic status and ABI data. Single regression analysis was used to show the correlation between the difference in upper-limb mean BP and the difference in SVR and the correlation between the difference in PG of the upper limb and diseased lower limb before and after EVT. Tukey’s honest significant difference test was used for comparison based on Fontaine classification and TASC II classification. P-values <0.05 were considered statistically significant. Statistical analyses were performed using a standard statistical program package (JMP 12; SAS Institute, Cary, NC, USA).

## Results

### Patient and lesion characteristics

The baseline patient characteristics are shown in **Table 1a**, and the lesion characteristics are shown in **Table 1b**. During the study period, 88 lesions of 56 patients were determined by AESCULON mini® data and treated with EVT. Sixty-five (73%) lesions had intermittent claudication, and 23 (26%) were afflicted with critical limb ischemia. Sixty-four (73%) lesions were found in the femoral-popliteal area, and 33 (38%) had chronic total occlusion. Fifty-five (98%) patients had hypertension.

**Table 1a:** Table 1 (a) Patient characteristics

	n=56
Age (years)	72.0±9.1 (48–90)
Sex (male)	39 (70%)
Height (cm)	159.9±8.4
Body weight (kg)	59.2±12.1
Body mass index (kg/m^2^)	23.0±3.2
Risk factors	
Current smoker	36 (64%)
Past smoker	10 (18%)
Hypertension	55 (98%)
Diabetes mellitus	37 (66%)
Dyslipidemia	34 (61%)
Chronic kidney disease	41 (73%)
Hemodialysis	7 (13%)
Coronary artery disease	33 (59%)
Cerebral vascular disease	13 (23%)
Medication	
Aspirin	44 (79%)
Thienopyridine	40 (71%)
Cilostazol	33 (59%)
Warfarin	3 (5%)
Direct oral anticoagulant	6 (11%)
Calcium channel blocker	31 (55%)
Angiotensin-converting enzyme inhibitor/Angiotensin II receptor blocker	36 (64%)
Beta-blocker	32 (57%)
Alpha-blocker	1 (2%)
Statin	38 (68%)
Laboratory data	
White blood cell (/µL)	6467.9±1671.3
Hemoglobin (g/dL)	12.2±1.6
Platelet (10^4^/µL)	21.7±6.1
Albumin (g/dL)	3.9±0.5
Blood urea nitrogen (mg/dL)	18.9 [15.8–25.1]
Creatinine (mg/dL)	1.07 [0.89–1.34]
eGFR (mL/min/1.73 m^2^)	45.7±20.9
Uric acid (mg/dL)	6.4±2.3
Total cholesterol (mg/dL)	171.9±37.7
High-density lipoprotein cholesterol (mg/dL)	45.4±13.9
Low-density lipoprotein cholesterol (mg/dL)	93.1±30.1
Triglyceride (mg/dL)	141.0±57.4
Hemoglobin A1c (%)	6.7±1.0
Brain natriuretic peptide (pg/mL)	95.3 [30.1–202.9]
Ultrasound cardiogram	
Left ventricular ejection fraction (%)	59.9±11.9

Values are presented as mean±standard deviation or median [range or 25%–75% interquartile range]. eGFR: estimated glomerular filtration rate

**Table 1b:** Table 1 (b) Lesion characteristics

	n=88
Disease severity (Fontaine classification)	
I	0 (0%)
IIa	40 (45%)
IIb	25 (28%)
III	3 (3%)
IV	20 (23%)
Lesion area	
Aortoiliac	24 (27%)
Femoral-popliteal	64 (73%)
Lesion position	
Right	46 (52%)
Left	40 (45%)
Bilateral	1 (2%)
Lesion length	
Aortoiliac (mm)	38.3±30.3
Femoral-popliteal (mm)	118.5±89.0
TASC II classification	
Aortoiliac	
A	13 (54%)
B	9 (38%)
C	0 (0%)
D	2 (8%)
Femoral-popliteal	
A	20 (31%)
B	17 (27%)
C	22 (34%)
D	5 (8%)
Chronic total occlusion	33 (38%)
Restenosis	9 (10%)
Restenosis again	1 (1%)

Values are presented as n (%). TASC: Trans-Atlantic Inter-Society Consensus

### Changes in hemodynamic status during EVT

**Figure 1:**
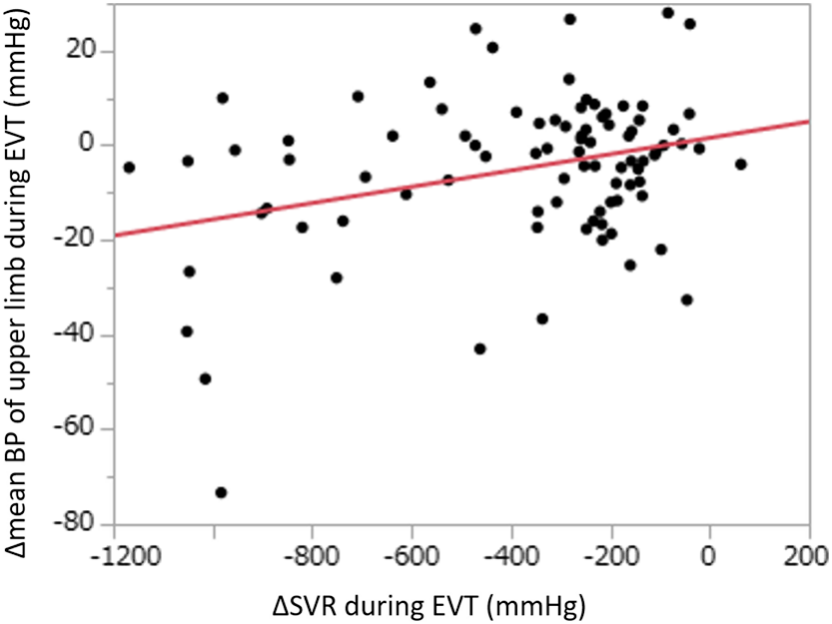
Fig. 1 Regression analysis of correlation between ΔSVR and Δmean BP in the upper limb during EVT (n=88).

**Table 2a** shows changes in hemodynamic status during EVT. Mean upper-limb systolic BP and SVR were high before EVT. Upper-limb systolic BP and mean BP significantly improved after EVT (from 157.7±30.9 mmHg to 144.8±26.9 mmHg, p<0.0001 and from 100.7±20.2 mmHg to 95.9±18.2 mmHg, p=0.0061, respectively). SVR significantly decreased from 2409.1±746.8 dynes·s·cm^−5^ to 2033.7±635.0 dynes·s·cm^−5^ (p<0.0001) after EVT. Similarly, cardiac output and stroke volume significantly increased after EVT. A positive correlation was confirmed in the single regression analysis of the difference in upper-limb mean BP and the difference in SVR before versus after EVT (**Fig. 1**). The difference in SVR before and after EVT was significantly greater in the Fontaine IV group than in the Fontaine IIa group (554.7±406.6 dynes·s·cm^−5^ vs. 312.9±245.7 dynes·s·cm^−5^, p=0.0151) (**Table 2b**).

**Table 2a:** Table 2 (a) Changes in hemodynamic status during EVT

n=88	Pre-EVT	Post-EVT	Change	p-value
Upper-limb systolic BP (mmHg)	157.7±30.9	144.8±26.9	−12.9	<0.0001
Upper-limb diastolic BP (mmHg)	72.2±17.4	71.4±16.7	−0.875	0.5297
Upper-limb mean BP (mmHg)	100.7±20.2	95.9±18.2	−4.9	0.0061
Heart rate (bpm)	75.0±17.1	74.7±17.5	−0.28	0.7023
Cardiac output (L/min)	3.6±0.8	3.9±1.0	0.3	<0.0001
Stroke volume (mL)	52.0±12.0	55.1±12.3	3.1	<0.0001
SVR (dynes·s·cm^−5^)	2409.1±746.8	2033.7±635.0	−375.4	<0.0001

Values are mean±standard deviation. p-values <0.05 were considered statistically significant. BP: blood pressure; EVT: endovascular treatment; SVR: systemic vascular resistance

**Table 2b:** Table 2 (b) Difference in SVR before and after EVT according to Fontaine classification

Fontaine classification	ΔSVR	p-value
IIa (n=40)	312.9±245.7	
IIb (n=25)	345.5±240.1	
III (n=3)	261.0±189.0	
IV (n=20)	554.7±406.6	
Fontaine classification		0.0198
IIa	IV	0.0151
IIb	IV	0.0804
III	IV	0.3573
IIa	III	0.9904
IIb	III	0.9631
IIa	IIb	0.9705

Values are mean±standard deviation. p-values <0.05 were considered statistically significant. ΔSVR indicates the difference in SVR before and after EVT. SVR: systemic vascular resistance

ΔSVR positively correlated with the Δmean BP in the upper limb during EVT (Y=1.5905995+0.0172355 X, R^2^=0.100546, p=0.0026). ΔSVR indicates the difference in SVR before and after EVT. Δmean BP indicates the difference in BP before and after EVT. BP: blood pressure; EVT: endovascular treatment; SVR: systemic vascular resistance

### Changes in BP before versus after EVT from ABI data

**Figure 2:**
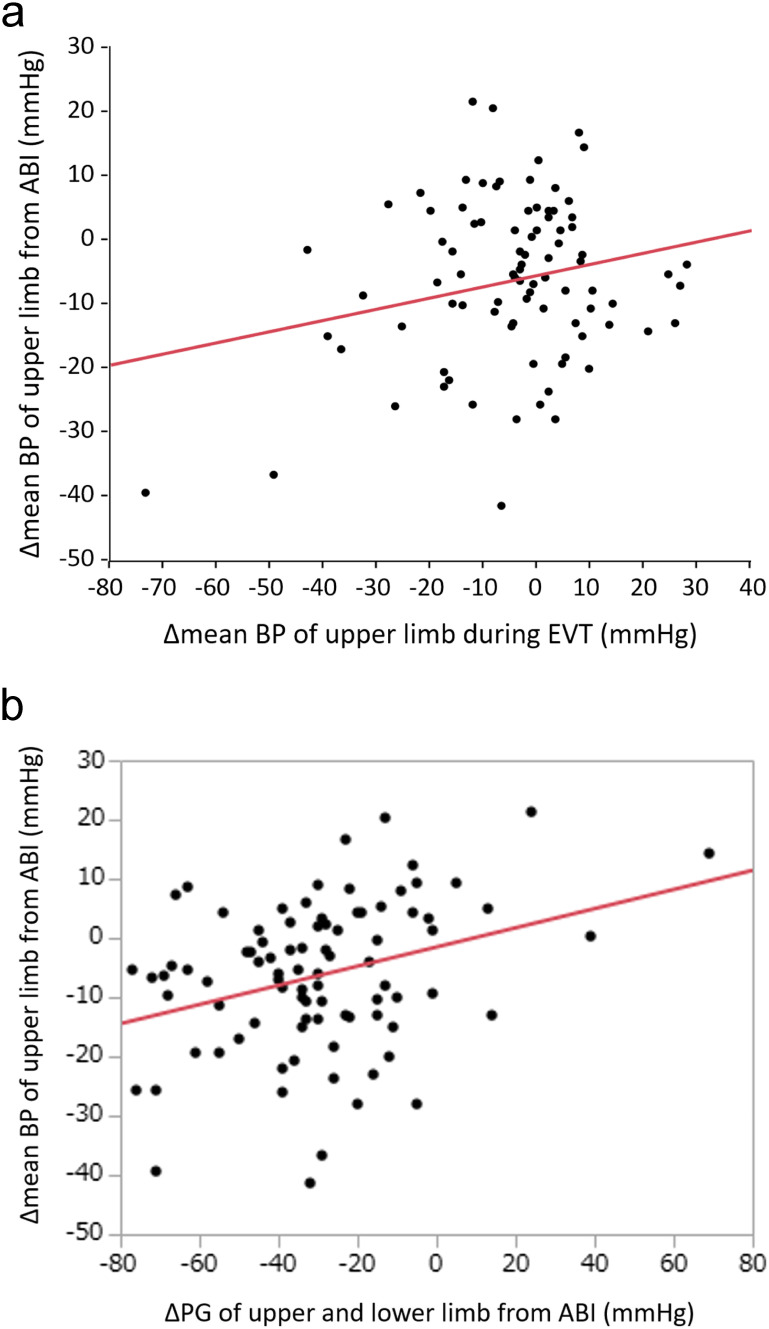
Fig. 2 (**a**) Regression analysis of correlation between Δmean BP in the upper limb during EVT and Δmean BP in the upper limb from ABI data before versus after EVT (n=88). Δmean BP of the upper limb from AESCULON mini® data positively correlated with Δmean BP of the upper limb from the ABI data (Y=−5.479292+0.1742755 X, R^2^=0.050297, p=0.0357). Δmean BP indicates the difference in BP after versus before EVT. (**b**) Regression analysis of correlation between ΔPG in the upper and diseased lower limb and Δmean BP in the upper limb from ABI data before versus after EVT (n=88).

**Table 3** shows changes in BP before versus after EVT using ABI data. Upper-limb systolic BP and mean BP significantly decreased after EVT (141.3±22.6 mmHg to 131.5±19.3 mmHg, p<0.0001 and 98.9±14.5 mmHg to 92.5±13.1, p<0.0001, respectively), and diastolic BP also significantly reduced. Diseased lower-limb systolic BP significantly increased from 87.1±24.9 mmHg to 107.2±27.9 mmHg after EVT (p<0.0001). Subsequently, ABI of the diseased side significantly improved from 0.62±0.17 to 0.79±0.20 (p<0.0001), and PG between the upper limb and the diseased lower limb significantly decreased from 54.2±24.5 mmHg to 24.3±28.2 (p<0.0001) after EVT. The difference in mean BP in the upper limb before versus after EVT from AESCULON mini® data positively correlated with that from the ABI data (**Fig. 2a**), whereas the difference in PG in the upper and the diseased lower limb positively correlated with the difference in mean BP in the upper limb before versus after EVT (**Fig. 2b**).

**Table 3:** Table 3 Changes in BP before versus after EVT using ABI data

n=88	Pre-EVT	Post-EVT	Change	p-value
Upper-limb systolic BP (mmHg)	141.3±22.6	131.5±19.3	−9.8	<0.0001
Upper-limb diastolic BP (mmHg)	77.5±12.3	73.0±12.1	−4.6	<0.0001
Upper-limb mean BP (mmHg)	98.8±14.5	92.5±13.1	−6.3	<0.0001
Diseased lower-limb systolic BP (mmHg)	87.1±24.9	107.2±27.9	20.0	<0.0001
Diseased lower-limb diastolic BP (mmHg)	58.0±17.1	59.8±13.4	2.2	0.2023
Heart rate (bpm)	75.4±18.7	76.8±18.0	0.7	0.3028
ABI of diseased side	0.62±0.17	0.79±0.20	0.17	<0.0001
PG between upper limb and diseased lower limb	54.2±24.5	24.3±28.2	−29.9	<0.0001

Values are mean±standard deviation. p-values <0.05 were considered statistically significant. ABI: ankle brachial index; BP: blood pressure; EVT: endovascular treatment; PG: pressure gradient

ΔPG in the upper and the diseased lower limb positively correlated with Δmean BP in the upper limb before and after EVT (Y=−1.491659+0.1619376 X, R^2^=0.103592, p=0.0022). Δmean BP indicates the difference in mean BP after versus before EVT. ΔPG indicates the difference in the PG in the upper and the diseased lower limb after versus before EVT. ABI: ankle brachial index; BP: blood pressure; EVT: endovascular treatment; PG: pressure gradient

## Discussion

The main findings of this study are as follows. First, EVT reduced upper-limb BP and SVR in patients with lower-limb PAD immediately after the procedure. Second, the difference in SVR was significantly greater in the Fontaine IV group than in the Fontaine IIa group. Third, the change in SVR positively correlated with the change in upper-limb BP during EVT. Fourth, the effect of BP reduction was also confirmed by ABI data before and after EVT. Fifth, the change in mean upper-limb BP during EVT positively correlated with the change in mean upper-limb BP using ABI data. Finally, the improvement in PG between the upper limb and the diseased lower limb by EVT was associated with a decreased upper-limb BP.

Based on lifestyle-related diseases, the prevalence of lower-limb PAD is increasing worldwide,^1,3)^ and the prognosis of patients with lower-limb PAD is poor due to complications such as coronary artery disease and cerebral vascular disease.^7,8)^ Hypertension, diabetes mellitus, dyslipidemia, chronic kidney disease, and smoking are considered important risk factors of lower-limb PAD.^9)^ Although hypertension is common in patients with PAD, PAD itself is an exacerbating factor of hypertension. Patients with PAD were more likely to have high brain natriuretic peptide levels^10)^ and left ventricular diastolic dysfunction^11)^ due to the involvement of high BP. In the mechanism of the progression of systemic arteriosclerosis, the arterial wall hardens by calcified plaque and develops stenosis. When the arteriosclerotic lesion reaches significant stenosis, the distal side becomes hypotensive and the proximal side (i.e., aorta and arteries of the upper limb) becomes hypertensive, that is, PG occurs. Subsequently, increased peripheral vascular resistance and hypertensive status occur. Pharmacological therapy is required for good BP control, and BP control can be resistant to multi-drug antihypertensive therapy in cases of severe organic stenosis.

Studies have demonstrated that EVT for patients with lower-limb PAD reduces upper-limb and central BP.^4,12)^ In studies using a multi-sensor catheter, Murgo et al. found that ascending aorta pressure was affected by forward pressure and backward pressure,^13)^ and that manual compression of bilateral iliac arteries increased ascending aorta pressure as backward pressure increased.^14)^ In other words, patients with lower-limb PAD are at a risk of exposure to high aortic pressure and upper-limb BP because the backward pressure is gradually increasing by the chronic progression of peripheral atherosclerotic lesions. Thus, the cancelation of significant arterial stenosis reduces systemic or upper-limb BP. In this study, we have shown that EVT could reduce PG at the stenotic/occlusive lesion, SVR, and systemic BP levels.

Fudim et al.^15)^ reported that central iliac arteriovenous anastomosis reduces the BP of patients with uncontrolled hypertension. Furthermore, Lobo et al.^16)^ highlighted that the hypotensive effect due to this device lasted until the chronic phase. In their trial (ROX CONTROL HTN Trial), office-based systolic BP decreased by a mean 25.1 mmHg and diastolic BP by 20.8 mmHg (p<0.0001 for both), whereas the mean 24 h ambulatory BP decreased by 12.6/15.3 mmHg at 12 months after central iliac arteriovenous anastomosis. These results suggest that arteriovenous anastomosis at the femoral vessel level reduced the peripheral vascular resistance and sustained hypotensive effect, even in patients with drug-resistant hypertension. Although the target patients in these studies were different from ours, the data were similar to ours in terms of the treatments that reduced peripheral vascular resistance. In our study, decreasing peripheral vascular resistance via EVT in patients with PAD led to decreased systolic and mean BP and improved lower-limb BP. Additionally, for patients with severe symptoms, the relief of leg pain may attenuate sympathetic activity and lead to a subsequent preferable effect on hemodynamic status. Jujo et al. recently reported that EVT may improve sensory disturbances associated with peripheral ischemic sensory neuropathy in patients with critical ischemia.^17)^ Lawes demonstrated that systolic BP and mean arterial pressure were associated with both fatal stroke and ischemic heart disease in the Asia–Pacific region.^18)^ Most importantly, EVT of the target lesions can lower peripheral vascular resistance, decrease BP, and reduce future cardiovascular events.^4)^

Our study has some limitations. This was a single-center retrospective study and not a randomized double-blind placebo-controlled trial. Furthermore, this study included a small sample size because the AESCULON mini® was used at the discretion of the attending physician. Also, AESCULON mini® was used instead of a reliable right heart catheter because it is noninvasive. Twenty-four-hour BP monitoring and AESCULON mini® data on days other than that of the EVT were lacking. EVT may be affected by sympathetic tension and sedation. The right atrial pressure used for SVR calculation was set to 5 mmHg, but an error may occur depending on the case. No significant difference was observed in SVR before and after EVT comparing chronic total occlusion and nonchronic total occlusion groups, iliac lesions, and femoral lesions. Furthermore, no significant difference was observed in SVR before and after EVT between TASC II classifications in both iliac and femoral lesions. It could be because the problem of vascular bed due to residual peripheral or contralateral lesions and the difference in the improvement rate of stenosis degree due to EVT were not considered. Finally, long-term outcomes were not evaluated in this study.

## Conclusion

The mechanism of BP reduction by EVT for patients with lower-limb PAD includes the reduction of SVR and PG between central BP and diseased lower-limb BP.
